# Angiogenesis in old-aged subjects after ischemic stroke: a cautionary note for investigators

**DOI:** 10.1186/2040-2384-2-26

**Published:** 2010-11-26

**Authors:** Eugen B Petcu, Robert A Smith, Rodica I Miroiu, Maria M Opris

**Affiliations:** 1 Griffith University School of Medicine, Gold Coast Campus, Griffith University, QLD 4222, Australia; 2 Universitatea Nationala de Educatie Fizica si Sport din Bucuresti, Facultatea de Kinetotherapie, 140 Constantin Noica Street, 060057 Bucuresti, Romania; 3 Universitatea de Medicină şi Farmacie Târgu Mureş, 38 Gh. Marinescu Street 540000 Târgu Mureş, Romania

## Abstract

Angiogenesis represents a form of neovascularisation of exceptional importance in numerous pathological conditions including stroke. In this context it is directly related to neuroregeneration which is seen in close proximity. However, numerous experimental data have been drawn from studies that have ignored the age criterion. This is extremely important as angiogenesis is different in young versus old subjects. Extrapolating data obtained from studies performed in young subjects or "in vitro" to old-age patients could lead to inexact conclusions since the dynamics of angiogenesis is age-dependent.

The current review covers the key features of brain senescence including morphological and functional changes related to the brain parenchyma, its vascular network and blood flow which could possibly influence the process of angiogenesis. This is followed by a description of post-stroke angiogenesis and its relationship to neuroregeneration and its modulation by vascular endothelial growth factor (VEGF) and insulin-like growth factor 1 (IGF 1), the most important factors active in old brain after ischemic injury.

## Introduction

Neovascularization represents a crucial phenomenon of paramount importance for the clinical outcome in various pathological conditions including cancer, myocardial infarction and cerebral stroke. Until not too long ago, it was thought that CNS damage induced by stroke is associated with irreversible tissue damage. However, recently, experimental data indicates that this is not the case and neuroregeneration is observed after stroke [1,2]. Interestingly, it appears that post-stroke neuroregeneration depends significantly on neovascularization which is encountered in several flavors: vasculogenesis, angiogenesis and arteriogenesis [3]. Vasculogenesis represents the embryological development of the blood vessels from angioblastic precursors and it has been recently described in patients after stroke [4]. Although, the extent to which vasculogenesis modulates post-stroke neuroregeneration is not known, the link between this process and angiogenesis is represented by VEGF and its receptor, which modulate transformation of immature precursor structures into mature capillaries [5]. Arteriogenesis is the growth of collateral arteries from pre-existing arterioles after blockage of the main artery. Compared with angiogenesis it does not require a hypoxic environment, and is typically activated by increased pressure and stress, such as that caused by occluded and partially occluded vessels [6]. The contribution of this process to neuroregeneration is unknown, however, numerous studies have reported that angiogenesis or formation of new capillaries from pre-existent vessels is closely related to neuroregeneration. After stroke, primordial cells capable to differentiate into functional neurons have been identified in the immediate vicinity of newly formed capillaries [7]. Therefore, it was claimed that this process can be regarded as a neurorestorative event promoting formation of new neurons from adult brain's own neural stem cells (NSC) [8,9]. Although factors such as matrix metalloproteinase-2 (MMP-2), matrix metalloproteinase-9 (MMP-9), tissue inhibitor of matrix metalloproteinase 1 (TIMP-1), Hepatocyte growth factor (HGF-alpha), monocyte chemo-attractant protein 1 (MCP-1) are increased after ischemic stroke the most important seems to be VEGF and its receptor which are increased in the periphery of the ischemic zone at 3 hours after stroke [10,11].

Although, a great deal of research has been performed, it seems that there are significant differences in the angiogenesis encountered in old subjects compared with young ones, including the extent of this process and the factors that may modulate it during different developmental stages. However, since angiogenesis depends on the pre-existing vascular network it is very important to understand if there is any relationship between what is happening with the brain *per se *and its cerebral blood vessels during senescence and the extent and/or modulation of post-stroke angiogenesis in this context.

The current review will highlight the most salient points related to the senescent brain and its vasculature, and then based on the published data we will review the factors which unequivocally modulate angiogenesis only in elderly subjects. This is necessary since ischemic stroke is mostly described in old age and extrapolating results obtained "in vitro" or from young subjects could lead to erroneous conclusions.

### CNS aging process: pivotal points

Normal aging is associated with a cognitive decline and understanding the related mechanisms remains a central challenge in neuroscience. Moreover, it is currently poorly understood how the CNS morphological changes associated with old age would affect the vascular network and ultimately post-stroke angiogenesis.

Morphological and physiological studies have tried to explain the decline in cognitive function associated with old age based on anatomical changes. More than fifty years ago, it was suggested that weight reduction in the senescent brain could be explained by a significant reduction in the neurons [12]. Recently, MRI studies conducted in healthy volunteers have revealed that brain weight loss may affect with predilection only some regions of the brain such as hippocampus or the prefrontal, frontal or enthorinal cortex and the loss of white matter is greater than the decrease in grey matter [13,14]. Other authors have recorded a reduction of the whole limbic system grey matter in healthy elderly [15].

Between 30 and 90 years of age, there is an overall decrease in weight of 14% recorded in the cerebral cortex associated with a 35% reduction in the hippocampus, and a 26% weight loss in the cerebral white matter [13]. However, it is still debatable if the changes seen in hippocampus are related to "normal" senescence as other studies have suggested that cell death and a decline in weight occurring in this region is not normally observed [16,17].

Studies in mice have indicated that aging may reduce both overall neural cell proliferation as well as the developmental pathway followed by proliferating cells in the brain, leading to fewer mature neurons replacing those lost to age or damage [18].

Regarding the vasculature, although earlier studies have shown no difference or even an increase in the cerebral arteriolar network in old versus young subjects, the current opinion is that old age is characterized by a significant decrease in density of vessels supplying the cerebral cortex compared with young controls as well as morphological changes of the remaining vessels [19-22]. More recently, Amenta et al (1995) have reported that the capillary network is significantly decreased in Wistar rats of 27 and 24 months versus young 12 month-old subjects [23]. Therefore, it seems unequivocal that aging is associated with a significant decrease in superficial cortical vessels which according to some authors, could be induced by an age-related decrease in growth hormone and insulin-like growth factor 1 [21]. However, growth hormone treatment administered to aged animals for 6 weeks did not alter hippocampal capillary density and did not ameliorate the age-related deficit in angiogenesis. Therefore, the exact relationship between growth hormone and angiogenesis remains to be elucidated. However, aged animals demonstrate a significant impairment in hypoxia-induced capillary angiogenesis compared to young animals [24].

Regarding the structural alterations of the vessels, significant aging-related microvascular degeneration was encountered especially in the periventricular white matter [25]. Also, in elderly rat subjects there is a decrease in smooth muscle and elastin in parallel with an increase in collagens which impair distension [26]. Senescent arterioles present with alterations in capillary endothelial cells including a thickened basement membrane, abnormal inclusions and abnormalities of the astrocytic endfeet [27-29]. However, the mechanism behind these changes remains poorly understood although it is accepted that these ultrastructural alterations could lead to a small leakage of blood in the parenchyma [30].

**Figure 1:**
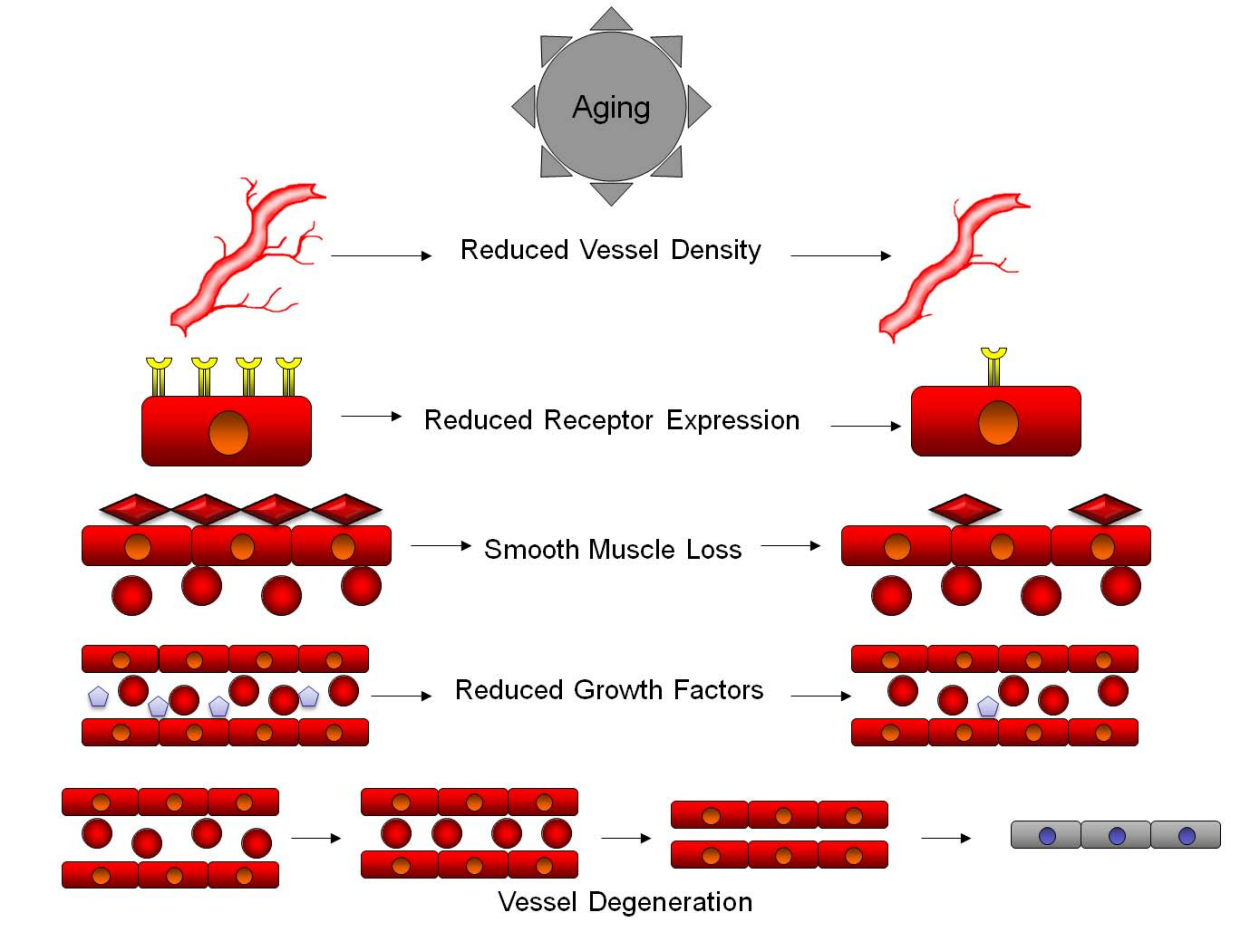
**Aging produces a number of physical and biochemical changes in the vascular system that contribute to the age related degeneration of the brain**. At the macroscopic level, reduced vessel density is observed in aging brains, leading to an overall reduced blood flow and oxygenation into brain tissue, that may be further exacerbated by pathological factors. At the cellular level, the vessel walls begin to reduce the expression of growth factor receptors, leading to reduced ability to respond to growth and survival factors. Vessels also begin to lose smooth muscle cells, reducing the capacity of vessels to maintain blood pressure, reducing oxygenation in certain circumstances. Aging also leads to a reduced expression of growth and survival factors into the bloodstream, further reducing the survival capacity of vessel lining cells. Many of these factors combine to lead to vessel degeneration, where reduced flow rates, reduced receptor expression and reduced growth factors lead to narrowing of vessels, further reducing flow and reducing vessel diameter, to a point where red blood cells can no longer flow through vessels, leading to loss of oxygenation of tissues and further degeneration into a string vessel state.

Regarding the cerebral blood flow in old-age, recent data suggests that it decreases with advancing age [31,32]. Previous studies have shown a direct correlation between cerebral blood flow and vascular density [33]. It seems that cerebral blood flow is modulated by circulating norepinephrine, NO and sympathetic noradrenergic innervations of cerebral arteries. The young subjects are characterized by little noradrenergic innervations of cerebral arteries and weak cerebral vasoconstriction. These innervations increase with age but decrease by middle-age in a murine model. However, the norepinephrine evoked cerebral vasoconstriction is stronger in mature and middle-aged rats and although the NO would limit the vasoconstriction, this is followed subsequently by a drop in cerebral blood flow [34]. Interestingly, previous studies conducted in a murine model suggest that a decreased blood flow is paralleled by a decline in cognitive tasks [35-37]. Therefore, one may speculate that a decrease in blood flow would lead to a decreased energetic support for neurons which complicates the functional profile of the aging brain including the post-stroke angiogenesis. The most important changes in vascularization with ageing in the brain are represented in Figure 1.

In addition, old age is characterized by the presence of several pathological entities affecting the vasculature. Cerebral atherosclerosis and small vessel disease are characterized by plasma protein infusion into the vessel wall, accumulation of foamy cells, and fibrosis while another relatively common condition, amyloid angiopathy, is associated with deposition of amyloid in the vessel wall [38]. Therefore, since the functionality of the nervous system is dependent upon a well developed and maintained blood supply, we could speculate that any morphological and physiological aging-related changes in the microvasculature could affect neuronal integrity. Related to Alzheimer's disease which is also seen in old-age patient, clinical studies have indicated that education has a protective effect. This has been attributed to a "reservoir effect", with the loss of neurons compensated for by more extensive neural connections. Given the links between dementias and reduced blood flow to the brain, it is possible that at least some of this effect may be mediated through a more developed vascular system, or potentially by retention of more juvenile modes of angiogenic response due to extended learning. This would make some sense in the context of experimental studies conducted in rats, which show that angiogenesis occurs in the adult rat cerebellum after physical exercise and motor skills learning [39].

**Figure 2:**
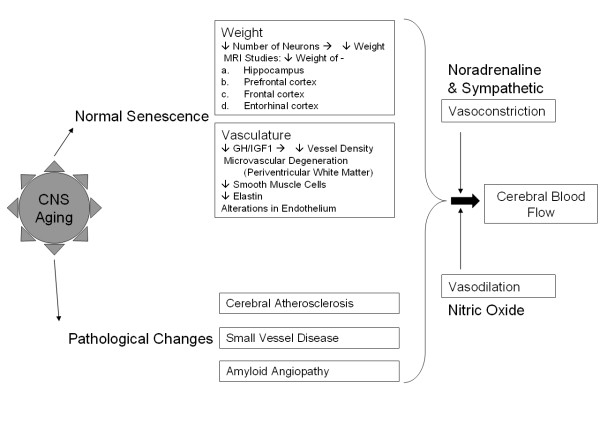
**Central nervous system "normal" senescence and pathological changes**. Aging in the CNS involves a range of diverse changes, including those associated with normal senescence and present in all surveyed brains. These include reduced weight of brain as a result of decreased neuron counts, as well as reduced vessel density and integrity in the vasculature. More pathological changes include atherosclerosis, small vessel disease and amyloid proteins. These combine with normal vaso- constriction and dilation effects to alter overall cerebral blood flow.

A brief account of central nervous system "normal" senescence and the pathological changes associated with it is presented in Figure 2.

### Post-stroke angiogenesis

One may define the process of angiogenesis as sprouting of new capillaries from pre-existent vessels. As a result of this ongoing process, the newly formed plexus increases gradually in size and remodels into a vascular network which ultimately forms endothelial-cell (EC) channels in close proximity with pericytes and smooth muscle cells. This cellular composition is of paramount importance for functionality of the new vessels including the strength of the wall and regulation of the blood flow [40]. Interestingly after stroke, neuroblasts which will further differentiate into fully functional neurons were identified in close proximity around the immature newly created vascular network [41]. This would suggest that neurogenesis depends on a preliminary angiogenesis.

Post-stroke cerebral angiogenesis represents an essential event of crucial importance that unfortunately is not completely understood. Several factors such as: beta-catenin, matrix metalloproteinase-2 (MMP-2) matrix metalloproteinase-9 (MMP-9), tissue inhibitor of matrix metalloproteinase-1 (TIMP-1), hepatocyte growth factor-alpha (HGF-alpha), monocyte chemoatractant protein-1 (MCP-1) and Angiopoietin1/Tie-2 as well as c-kit are increased after ischemic stroke [42].

Beta-catenin is a member of the cadherin complex and a signaling protein in the Wnt pathway. It has been linked to the proliferation of neuronal progenitor cells in stroke induced neurogenesis [43]. In stroke, MCP-1 is thought to be one of the major factors influencing infiltration of the infarct region by leukocytes and is linked with increased volume of the infarct and increased damage [44]. Experimental data suggests that in rats with middle cerebral artery occlusion the lack of MCP-1 or its receptor CCR-2 is associated with a significant decrease in the number of migrating neuroblasts reaching the ischemic area. This affects the neural regeneration negatively [45]

The MMP family participates in the breakdown of various extracellular matrix proteins, and is associated with wound healing and tissue remodeling. In stroke, the MMPs have been implicated in the breakdown of the blood-brain barrier and increased damage [46-51]. Different MMPs are expressed in different conditions and at different times following stroke, with MMP-2 being amongst the first activated, followed by MMP-9 in later stages of inflammation and repair [52].

Research conducted in murines recently, indicates that the major source of MMP-9 in cerebral ischemia is represented by bone-marrow derived cells [53]. Remarkably, MMP-9 promotes neural progenitor cells migration towards the ischemic brain area in a model of transgenic mice after photothrombotic ischemia [54].

Clinical studies conducted in patients with various types of stroke have revealed that MMP-2 gene is associated with the development of lacunar stroke [55]. However, it rises in parallel with MMP-9 in a rat model of transient cerebral ischemia [56]. It seems that MMP-2 and MMP-9 expression is decreased after minocycline administration in rats with induced cerebral ischemia. This is associated with a decreased incidence of hemorrhage and decreased degradation of collagen IV and laminin alpha in the brain. Overall, the neurological outcome in cerebral ischemia is improved by minocycline through MMP-2 and MMP-9 downregulation[57]. In patients with severe stroke both MMP-2 and MMP-9 have a strong association with edema formation and midline shift [58].

The TIMPs are inhibitors of the MMPs and their induced overexpression has been shown to aid in reduction of infarct size and recovery in a rat model of ischemia [59].

HGF-alpha is known to induce angiogenesis and has been seen to increase in expression following MCAO in mouse models, though a high serum level of the protein was found to be an independent risk factor for stroke in postmenopausal women [60,61].

Experimental evidence suggests that angiopoietin 1 (Ang1) and its endothelial kinase 2 (Tie2) are up-regulated after stroke by a nitric oxide donor and subsequently promote neuroblast cell migration towards the ischemic area [62]. Inceased levels of Ang1/Tie2 have been recorded in ischemic stroke models after simvastatin treatment and infusion of bone marrow stromal cells. Both treatments result ultimately in vascular stabilization and angiogenesis [63,64].

**Figure 3:**
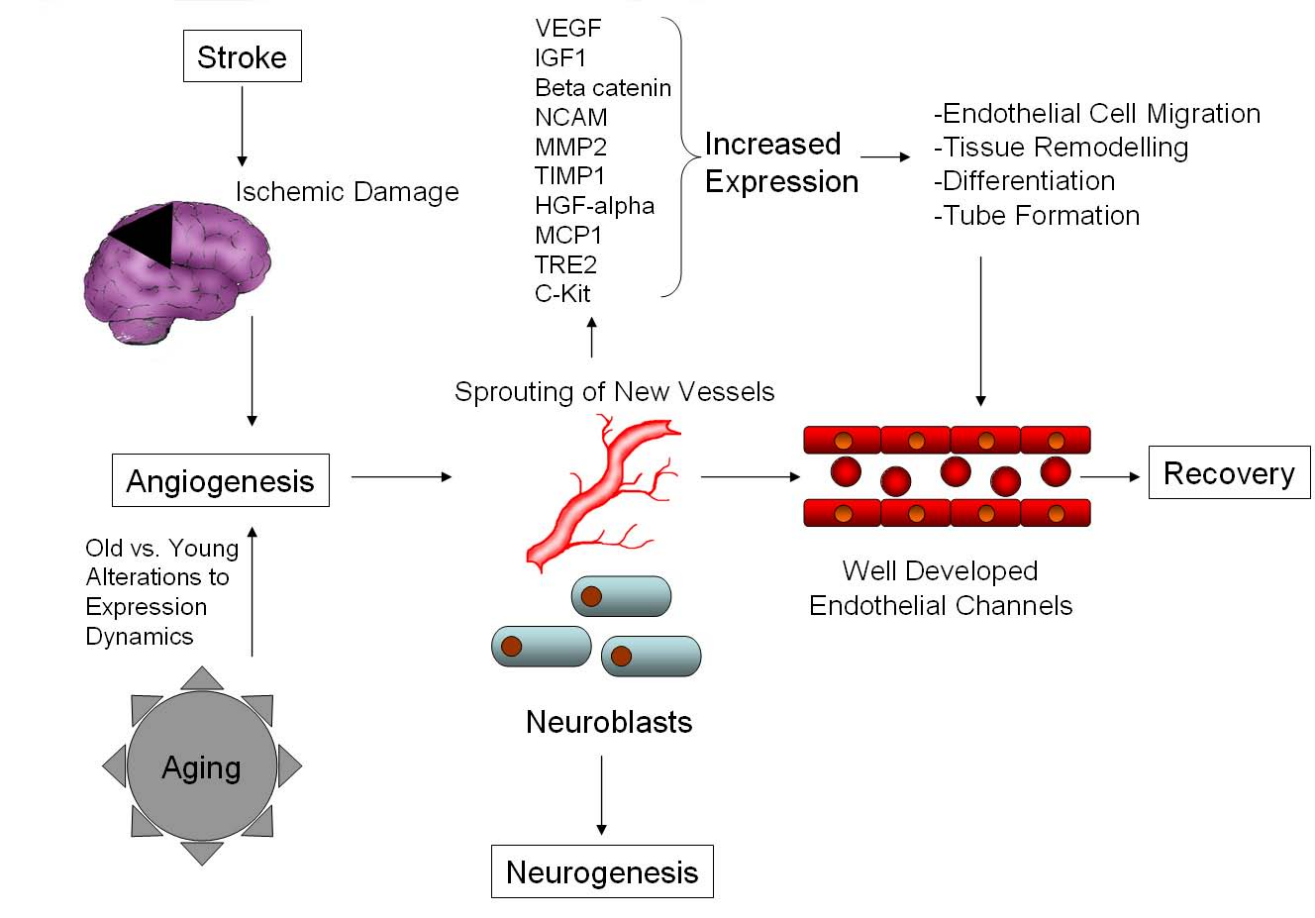
**Possible molecular mechanisms and general effects of angiogenesis after stroke in elderly subjects**. Stroke causes ischemic damage to the brain, activating angiogeneic mechanisms in response. The degree of response is modulated by changes to the neural and vascular response caused by aging, but the general response remains similar. Growth factors, tissue remodeling and inflammatory proteins are released, leading to the building of new, well developed endothelial channels, which aid recovery.

The above mentioned factors are involved in events of paramount importance namely, endothelial cell migration, tissue remodeling, differentiation and tube-formation, vessel stabilization and stem cell homing mechanisms in areas of revascularization. Therefore, we could speculate that therapy modulating their expression and the activity of these factors could help the recovery and restoration of the damage after stroke [42]. Several of these factors are also associated with arteriogenesis, and it is possible that multiple systems may come into play after stroke, depending on its severity, or that there is a certain amount of cross-talk between the angiogenic and arteriogenic systems. A concise summary of the possible molecular mechanisms and general effects of angiogenesis after stroke in elderly subjects is presented in Figure 3.

However, at the present time, we do not have any information on the dynamics of the above mentioned factors with aging. Since ischemic stroke is most likely to be encountered in elderly, any possible therapy should be verified in the context of aging. This means taking into consideration the level of the targeted factors in old age, the ability of the vascular and support systems to respond to them and the integrity of downstream gene expression in the targeted pathways. To date, only VEGF and IGF-1 have been evaluated as modulators of neovascularisation in old subjects.

### Post-stroke angiogenesis modulators in old age subjects

#### VEGF and its receptors

Without any doubt, vascular endothelial growth factor (VEGF) is the most important promoter of angiogenesis secreted by endothelial cells and pericytes [65].

Recent research evaluating the role of remnants of capillaries the so-called string vessels, indicates that VEGF represents an endothelial cells survival factor. String vessels have been described in ischemic conditions but also in normal human brains. They represent capillaries that have lost their endothelial cells. It seems that their presence is associated with an age-related decline in VEGF [66].

One of essential aspects related to VEGF is represented by the fact that neurogenesis takes place near cerebral capillaries where both VEGF and angiogenesis are up-regulated. Therefore, it is believed that that VEGF links neurogenesis and angiogenesis [67-69]. After ischemic stroke, VEGF is detected on microglial cells and macrophages and also on capillaries within the peri-ischemic zone, where a pro-angiogenic effect may be identified. Notably both VEGF mRNA and one of its receptors (VEGFR-1) are increased in the periphery of the ischemic zone at 3 hours after stroke reaching a peak after 24 h, and remaining detectable 7 days post stroke [70]. Therefore, it was argued that an increased production of VEGF and its receptor represent a physiological response to ischemia which ultimately aims to preserve and to restore the damaged nervous tissue, consistent with the functions of the VEGF system in other tissues.

Remarkably, experiments conducted in a murine model of middle cerebral artery occlusion have indicated that apoptosis of the neurons in the penumbra is significantly depressed after the beginning of angiogenesis and it appears that anti-apoptosis is achieved by VEGF during angiogenesis via the induced expression of survivin in endothelial cells [70,71].

Most importantly, the experimental data on VEGF has been positively matched by clinical data. Neurons, endothelial cells, and astrocytes in the penumbra from patients that died after ischemic stroke are characterized by an increased expression of VEGF compared with contralateral areas [72]. Moreover, in patients with acute ischemic stroke serial measurements pointed towards an increased serum level of VEGF, which correlated very well with infarct volume and clinical disability [73].

In atherosclerotic lesions VEGF is induced by C-reactive protein (CRP) and significant time-dependent up-regulation of VEGF-A mRNA expression and its protein was recorded in monocytes after treatment with CRP. It seems that the most plausible mechanism for this is represented by the activation of a PI3-kinase and an extracellular signal regulated kinase (ERK) [74,75]. Also, VEGF modulates hypoxia-induced CNS angiogenesis [76,77]. In middle cerebral artery occlusion (MCAO) experiments, the induced hypoxia represents a signal for activation of hypoxia-inducible factors which subsequently promote expression of VEGF and its receptor genes [78]. However, as mentioned above, hypoxia-inducible angiogenesis is decreased with aging [24]. Therefore one may conclude that VEGF and/or its receptors are down-regulated with aging, and/or that long term effects, such as promoter methylation retard the capacity of aged cells to respond to pro-angiogenic signals brought on by hypoxia. Interestingly, in selected areas of brain such as the hippocampus there is a significant decrease of VEGF between young and middle age animals. However, there is no difference between middle age and elderly subjects [65,79]. It appears that decreased hippocampal VEGF concentration in middle age is related to numerous changes in the cellular substrates for VEGF. These abnormalities include reduced proliferation of endothelial cells in capillaries and degeneration of endothelial cells, especially in the vascular niche surrounding stem/progenitor cells [67].

Recently, in vitro and in vivo studies conducted by Emerich et al (2007) have shown that choroid plexus epithelial cells from young subjects secrete more VEGF and are metabolically more active than the same type of cells from aged animals [80]. Moreover, transplantation of choroid plexus epithelial cells from old subjects is significantly less neuroprotective than the transplant of the same type of cells from young rats [80]. In addition, the angiogenic and neurogenic response to adeno-associated viral vector-VEGF injections is decreased in the aged mouse brain [81]. Brains from 24 month old mice, compared to those of 3 and 12 month old mice, in the study by Gao *et al*. showed reduced expression of VEGFR-2, another VEGF receptor commonly associated with angiogenic functions [81]. This may explain the reduced angiogenic response seen in these brains. In addition, the brains of 24 month old mice showed lower levels of neuroprogenitor cells following stroke and VEGF stimulation. Thus, it may be that reductions in VEGF receptor expression, accumulation of senescence markers and other metabolic changes in endothelial and neural cells lead to reduced proliferative response.

Since the activity of other pro-angiogenic factors such as placental growth factor (PLGF) and neuropilins (NP1 and NP2) is directly linked to the level of VEGF/VEGF receptors, one may speculate that their level could also be affected by the aging process [82-84]. However, no studies have been specifically designed to evaluate these factors in aged subjects.

**Figure 4:**
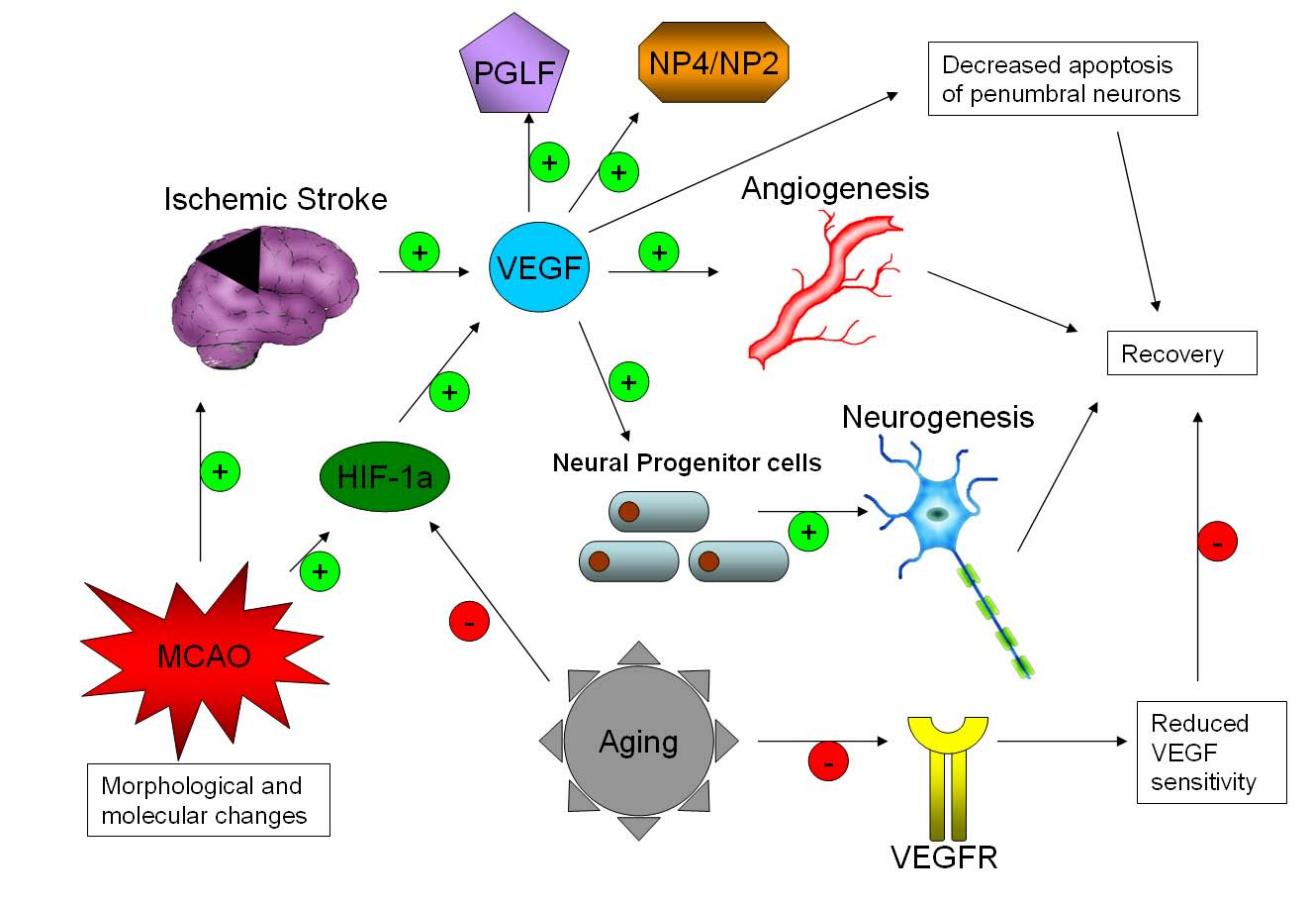
**VEGF and post-stroke angiogenesis in elderly subjects**. Morphological and molecular changes lead to medial cerebral artery occulsion and similar states that give rise to full ischemic stroke. Both occulsion and outright stroke lead to stimulation of VEGF release by affected cells, which drives the expression of additional effector proteins, increases angiogenesis, promotes cell survival and neurogenesis, leading to recovery. Aging reduces the activation of certain responses, including HIF-1a and the ability of cells to respond to VEGF through downregulation of receptors, impairing repair and recovery.

A summary of the possible VEGF related mechanisms of post-stroke angiogenesis in elderly subjects is represented in Figure 4.

#### Insulin-like growth factor I (IGF-1)

Experiments conducted on human tissue have shown that increased levels of insulin-like growth factor I (IGF-I) and vascular endothelial growth factor (VEGF) are associated with neovascularization induced by retinal ischemia [85].

Anderson et al (2002) have suggested that IGF-1 induces neurogenesis from adult-derived neural precursors in vitro and has similar effects on the hippocampal progenitor population in vivo [86]. Relatively recent research has proved that insulin-like growth factor-I (IGF-I) may regulate neurogenesis in the aging brain. This experimental hypothesis has been derived from the fact that IGF-1 influences neuronal production during development and, similar to neurogenesis itself, it decreases with old age. Lichtenwalner et al (2001) have showed an age-dependent reduction in the number of newly generated cells in the adult dentate subgranular proliferative zone [87]. This was paralleled by a 60% reduction in the differentiation of newborn cells into neurons. Remarkably, restoration of IGF-I levels in senescent rats significantly restored neurogenesis, with an approximately three-fold increase in neuronal production reported. Therefore, the authors suggest that IGF-I may significantly modulate neurogenesis in aging hippocampus and that an age-related decline in IGF-I-dependent neurogenesis could contribute to age-related cognitive changes [87].

Experimental studies conducted in murine models have shown that vasculature and meninges are an important source of IGF-1 for the brain and that expression continues throughout life. In addition, there are no changes in IGF-1 gene expression with age but IGF-1 protein levels decrease suggesting that translational deficiencies or deficits in the transport of IGF-1 through the blood-brain barrier contribute to the decline in brain IGF-1 with age. This would imply that aging is correlated with significant changes in the IGF-1 axis which cause the brain senescence itself [88].

As mentioned previously, the vascular density on the surface of the cortex decreases with aging. This correlates with low IGF-1 plasma levels, which would suggest that IGF-1 has an important role in the decline of vascular density associated with aging [21]. In the hippocampus, concentrations of IGF-1 decrease significantly between young and middle aged experimental animals. The drop in IGF-1 levels is also recorded in old-age subjects. However, there is no significant difference between IGF-1 levels recorded in middle-age and elderly subjects [89].

Recent experimental research has revealed that both local and serum IGF-1 modulate angiogenesis after brain trauma. Low IGF-1 level impairs angiogenesis and promotes vascular dysfunction [90]. These results confirm previous data from elderly patients with ischemic stroke which have reported up-regulated levels of IGF-1 in the context of a well established angiogenesis [91]. Interestingly in a study conducted in elderly patients with stroke, serum IGF-1 levels assessed within 24 hours of the onset of stroke were significantly lower than levels in controls. Presumably these patients had low levels of IGF-1 before stroke, though this has not been established. However, low levels of IGF-1 were frequently associated with death [92]. Other studies have shown that high levels of IGF-1 *before *stroke are associated with a larger ischemic area [92,93]. The authors speculate that a high level of IGF-1 would increase the susceptibility to ischemia produced by middle cerebral artery occlusion. In this context the damage induced by increased IGF-1 could be produced by activation of MAPK [94]. Moreover in an experimental setting, IGF-1 is beneficial if given *after *ischemia but not before the event [95-97]. Unfortunately, we do not have a clear explanation of these facts yet, though it may be that high levels of IGF-1 prior to stroke may reduce tissue responsiveness afterwards, due to negative feedback loops reducing expression of downstream signal receptors. It is also possible that a high level of IGF-1 prior to stroke may be indicative of substantial, but low-level hypoxia in the tissue and that the already stressed neurons are less likely to survive an ischemic event. Overall, the role of IGF-1 in modulating angiogenesis and vascular development is still not completely elucidated. Further research should take into consideration not only the age of the subjects but also the dynamics of IGF-1 level before, during and after ischemic damage produced by stroke.

#### Other growth factors and cellular studies

Although VEGF and IGF seem to be the main actors in post stroke angiogenesis, there are a number of other factors in play. As well as the relationship between gene expression and age in the VEGF and IGF systems, similar relationships have been noted for fibroblast growth factor (FGF) and transforming growth factor beta 1 (TGFb-1).

As for VEGF, expression of FGF is increased in the area surrounding a stroke in human subjects [98].

In the rat and porcine models, however, an age specific reduction in FGF receptor expression and signaling has been observed, and it seems likely that a similar loss of expression is experienced in human brains [98-100].

Likewise, TGFb-1 is more highly expressed in the immediate area around a stroke, but is not sufficiently increased to affect measurements of concentration [73].

Studies in cell cultures have indicated that long term cultured cells show reduced capacity to form tubes in appropriate culture conditions, as well as increased morphological heterogeneity [101]. Cell culture models of older cells also show similar losses of VEGF expression to those observed in older whole animals [79]. This would indicate that in both *in vivo *and *in vitro *studies, there are significant alterations in the behavior of aged endothelial cells compared to younger ones.

### In conclusion

Old age is associated with a variety of morphological, physiological and metabolic features which may alter recovery after stroke. These features combine normal aging of brain with the ongoing processes of neurodegeneration and small vessel disease. These work in synergy to reduce the capacity of both neural and vascular cells to respond to ischaemic insult, as well as compromising their maintenance and basic functions.

One of the most important changes is represented by a decrease of the normal cerebral vascular network. This may affect the ability of the old-age subjects to mount an adequate pro-angiogenic response after an insult such as stroke. VEGF/VEGFR and IGF-1 represent the most important modulators of post ischemic angiogenesis. Research is showing that some of the changes to the mechanisms behind these effects are similar in both neural and vascular tissues. However, we cannot be sure if the decrease in their level is due to morphological, physiopathological or metabolic changes, or how these may interact to contribute to the changes seen in the aging brain and vasculature. The role and the promoters of these factors are also incompletely understood, but they may be related to age-associated loss of cellular proliferative ability and increasing negative regulation by cellular senescence markers. In addition, since ischemic stroke is a pathological entity most likely encountered in the elderly, future studies should evaluate any possible therapeutic targets related to post ischemic stroke angiogenesis only in aged animals or higher passage *in vitro *systems. More research is also needed to fully explain the mechanisms at work in the ischemic senescent brain which may influence angiogenesis and/or the final outcome of the subject. This is crucial not only to understand the nature of stroke, but also to provide the best therapeutic response with current methods and to develop new treatment and, potentially, preventative options.

## Competing interests

In the past five years, *the authors of this article have not *received reimbursements, fees, funding, or salary from an organization that may in any way gain or lose financially from the publication of this manuscript, either now or in the future.

*The authors of this article *do not hold any stocks or shares in an organization that may in any way gain or lose financially from the publication of this manuscript, either now or in the future

*The authors of this article *do not hold or are currently applying for any patents relating to the content of the manuscript.

*The authors of this article *have not received reimbursements, fees, funding, or salary from an organization that holds or has applied for patents relating to the content of the manuscript.

*The authors of this article *have no other financial competing interests.

## Authors' contributions

EBP has made substantial contributions to conception, design, and interpretation of data for this study including the drafting of the manuscript and revising it critically for important intellectual content and has given the final approval for the version to be published.

RAS has made substantial contributions to interpretation of data for this study including the drafting of the manuscript and its figures revising it critically for important intellectual content and has given the final approval for the version to be published.

RIM has made substantial contributions to conception and interpretation of data for this study including the drafting of the manuscript and revising it critically for important intellectual content and has given the final approval for the version to be published

MMO has made substantial contributions to interpretation of data for this study including the drafting of the manuscript and revising it critically for important intellectual content and has given the final approval for the version to be published.

## Authors' information

EBP, MD, MHSc (Pharmacology), PhD (Neurobiology) is an academic anatomic pathologist, currently Senior Lecturer with training and experience in Neurobiology research focusing on molecular mechanisms of recovery after ischemic stroke. He conducts a newly created neurobiology research group at his institution.

RAS, BSc (Hons), PhD (Mol Biology) is a scientist conducting angiogenesis research. Particularly he is interested in molecular factors modulating angiogenesis in various settings such as stroke and cancer. He is a Post-Doctoral Fellow and co-supervises several graduate students.

RIM, MD, PhD (Neurorehabilitation) is an academic physician with special interests in research in Neuroanatomy and Neurorehabilitation after stroke including the potential role of modulators of angiogenesis in this pathological entity. She is the head of her department where she currently works.

MMO, MD, PhD (Cardiology) is an academic physician interested in angiogenesis modulation in several pathological entities including atherosclerotic ischemic stroke and cardiovascular disease. She is the section head in her academic institution.
